# Comparison of Locked Plates and Blade Plates for Varus Osteotomy of the Proximal Femur by the Finite Element Method

**DOI:** 10.1055/s-0043-1775889

**Published:** 2024-03-21

**Authors:** Wilisson Ribeiro Filho, Eduardo Henrique Silva Wolf, Claudio Santili, Miguel Akari, Vanessa Guimarães de Freitas, Leonel Vieira Doudement

**Affiliations:** 1 Grupo de Ortopedia e Traumatologia Pediátrica, Pontifícia Universidade Católica de Campinas, Campinas, SP, Brasil; 2 Programa de Pós-Graduação em Ciência e Engenharia de Materiais, Universidade Federal de São Carlos, São Carlos, SP, Brasil; 3 Grupo de Ortopedia e Traumatologia Pediátrica, Faculdade de Ciências Médicas, Santa Casa de São Paulo, São Paulo, SP, Brasil; 4 Ortopedia Pediátrica, Pontifícia Universidade Católica de Campinas, Campinas, SP, Brasil

**Keywords:** orthopedics, traumatology, femur, bone plates

## Abstract

**Objective:** The present study compared the difference in load and pressure distribution behavior of the blade plate and locked plate for varus osteotomy of the proximal femur per the finite element method.

**Methods:** Modeling was performed by scanning a medium-sized left femur with medial valgus deformity made of polyurethane.

**Results:** The stiffness of the locked plate is higher compared with that of the blade plate. However, this difference was not significant. In addition, the locked plate has proximal locking screws to ensure that the bending moments on the screws are smaller during loading.

**Conclusion:** In summary, both plates are well-established and effective. However, the study using the finite element method plays a fundamental role in understanding the load and pressure distribution of the implant. Moreover, it opens up new possibilities for further studies, including surgical proposals and customized implant materials.

## Introduction

Varus osteotomies of the proximal femur are pediatric reconstructive surgeries widely performed in patients with neurological abnormalities, congenital hip diseases, sequelae, and acquired conditions. 1 2 Fixation of these osteotomies may use several implants, including blade plates, dynamic compression plates (DCP), locked plates for the proximal femur, unilateral and circular external fixators, Kirschner wires, and screws. 1 2 3 The surgical complexity of these procedures resulted in advanced synthesis materials to facilitate surgery and improve outcomes. 1 2 4

The most used materials in varus osteotomy of the proximal femur include locked and blade plates. 1 2 3 4 Clinically, blade and locked plates have no statistical difference in the risk of failure (breakage), 3 which is the worst complication related to the choice of implant. Biomechanical tests with load application in experimental models show that locked plates with a support screw have higher axial resistance, lower resistance to torsion, and irreversible equivalent strain to deforming cycles compared with blade plates. 4

The finite element method (FEM) is a mathematical tool used to solve problems in engineering because it explores the effects of load application on the bone and its biomechanical behavior. One of its main advantages is its potential use in solids with irregular geometry presenting heterogeneous material properties. The introduction of FEM in orthopedic biomechanics occurred in the 1970s; since then, the number of publications on load analysis in bones, arthroplasty, and osteosynthesis has been increasing. 5

The present study aims to analyze, using FEM, the load and pressure distribution behavior when implanting blade or locked plates for varus osteotomy of the proximal femur.

## Methods

### Model Generation

**Fig. 1:**
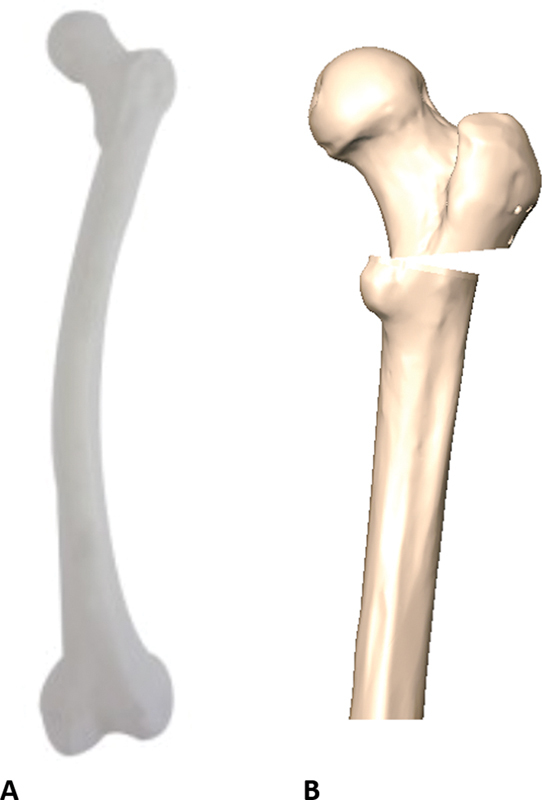
Valgus femur from Nacional Ossos for scanning. ( **A** ) Simplified femur and ( **B** ) varus osteotomy. Source: Data constructed by the authors using SolidWorks software.

Modeling was based on a computed tomography scanning of a left femur made from medium-sized polyurethane (Nacional Ossos, Brazil, reference number 2025 DMVL) and presenting a medial valgus deformity. The software used for plate scanning and modeling was SolidWorks (Dassault Systems SolidWorks Corp., Waltham, MA, USA) ( Fig. 1a ).

For femur simplification, we sectioned the diaphysis and discarded the distal part since it would not be studied. A varus osteotomy was performed for 20° correction. This simplification provided a gain in calculation processing by FEM without result distortion. 6 The environment (femur) was the same for both studies, not distorting or favoring any data ( Fig. 1b ).

**Fig. 2:**
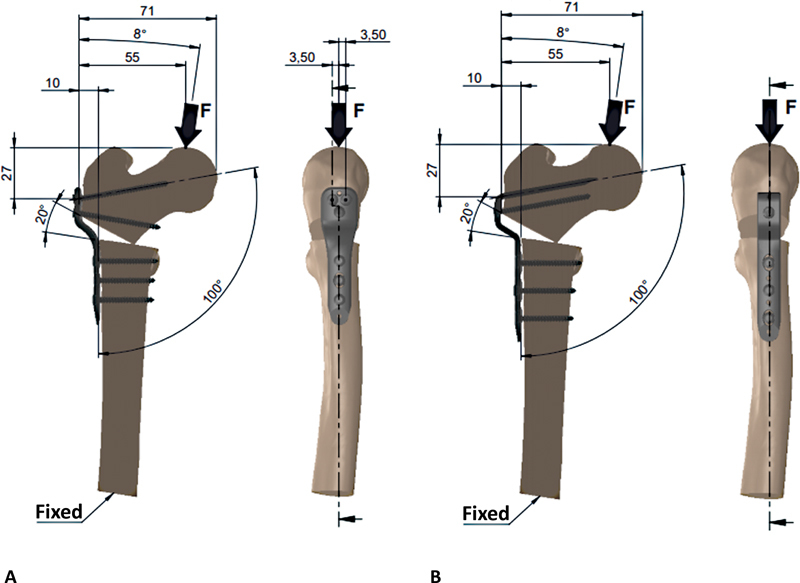
Locked plate fixed to the femur with screws ( **A** ). Blade plate fixed to the femur with screws ( **B** ). Source: Data constructed by the authors using SolidWorks software.

Fixation of the first femur employed a locked plate for the proximal femur with a 10-mm step, 100°, and three holes (Techimport, Rio Claro, SP, Brazil, reference number TI030.1003.100). Proximal fragment fixation used 3.5 × 50-mm diameter locked screws in two holes and a 3.5 × 40-mm locked screw in the third hole. Distal fragment fixation used 1 3.5 × 30-mm and 2 3.5 × 30-mm locked screws ( Fig. 2a ). Fixation of the second femur employed a blade plate for the proximal femur with a 10-mm step, 100°, 3 holes, 50-mm blade (Techimport, Rio Claro, SP, Brazil, reference number ref. TI030.1010.350). The proximal hole received 3.5 × 50 mm-diameter locked screws, while the distal non-locked plate holes received two 3.5 × 30-mm non-locked compression screws and a 3.5 × 30-mm locked screw for distal fixation ( Fig. 2b ).

Plate and screw assembly through visual positioning used the SolidWorks software (Dassault Systems SolidWorks Corp., Waltham, MA, USA). We created a 27-mm distance restriction between the proximal axis of the locked plate screw and the central axis of the blade plate and a 71-mm distance restriction between the medial face of the proximal femur and the inner face of the plates. These restrictions standardized the flexor moment generated by applying the axial force and the displacement stress assessment ( Fig. 2 ).

### Material Properties

Plates, screws, cortical, and cancellous bone models were homogeneous, linear, elastic, and isotropic, based on the properties described in the literature ( Table 1 ). The yield stress was set at 795 MPa, the limit stress of the elastic region of the titanium alloy. 7 8

**Table 1:** Elastic modulus, Poisson ratio, and number of fatigue cycles for all materials (data from Maurer et al., 1999 5 and Janecek et al., 2015 6 )

Materials	Elastic modulus (E) [MPa]	Poisson ratio	Yield stress at 10 ^6^ cycles [MPa]
Cortical bone	8,700	0.33	200
Cancellous bone	500	0.30	125
Titanium alloy	110,000	0.34	540

Source: Data obtained by the authors.

### Simulation Parameters (Load, Mesh, and Contact Conditions)

Fig. 2 shows the applied forces of 450, 500, 550, and 600 N considered a normal loading position, which assumes that the load vector has an angle of 8° of adduction with the hip longitudinal axis in the plane. 6 9 10 11 12 13 14 15 16 17 In a clinical situation, both values are greater than those produced by touch support with crutches and should provide sufficient postoperative stability. 10 11

The force application frequency was 1 Hz, considering walking 1 step per second. 11 12 Since this is a temporary fixation device, the plate must withstand at least 10 ^6^ cycles, equivalent to ∼ 1 year, with this frequency. 13 We constrained the transverse face of the femoral shaft in all translational degrees of freedom ( Fig. 2 ).

**Fig. 3:**
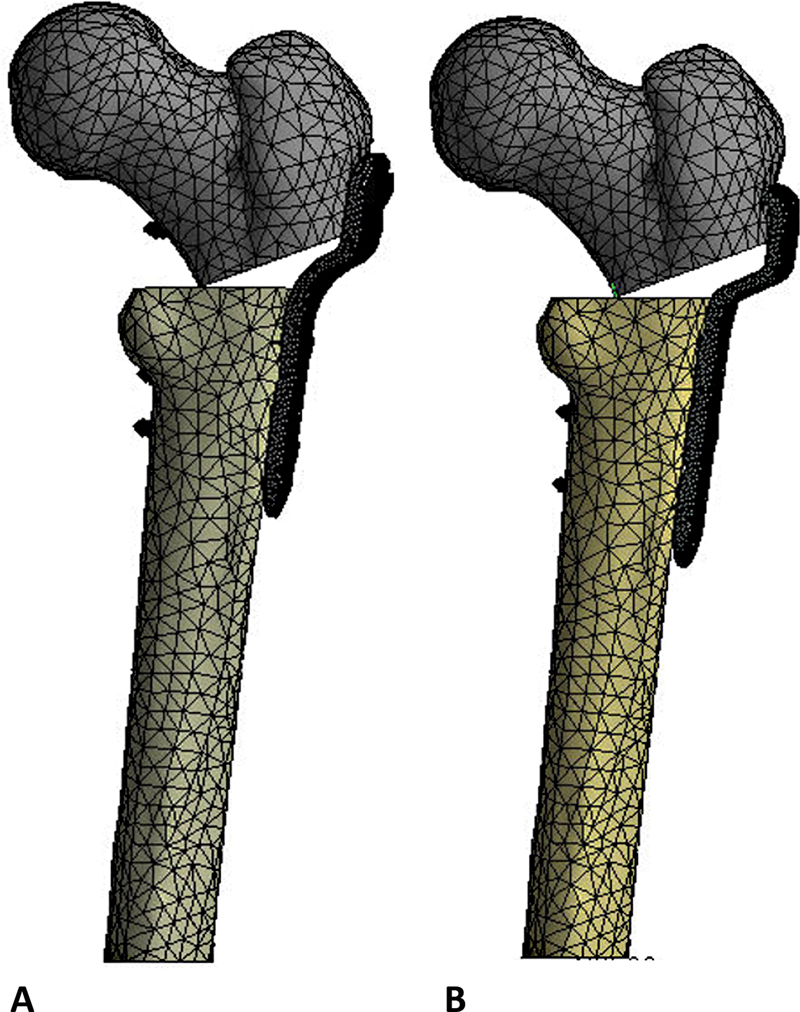
Mesh applied to three-dimensional locked plate models ( **A** ). Mesh applied to the three-dimensional blade plate models ( **B** ). Source: Data constructed by the authors using SolidWorks software.

We merged the mesh models with three-dimensional quadratic tetrahedral elements in SolidWorks software of 1 mm for plates, 0.5 mm for screws, and 5 mm for bone. 14 Bonding contact occurred between bone tissue and implants, except for osteotomy interfaces ( Fig. 3 ).

Plate and screw contact surfaces had a friction coefficient of 0.34. 15 17 The screw and plate contacted at the surface of the screw head and the surface of the countersunk in the plate holes, all with a restriction to not allow penetration between them. As for the contacts, locked screws in plate holes were deemed connected and fixed. 17

## Results

**Fig. 4:**
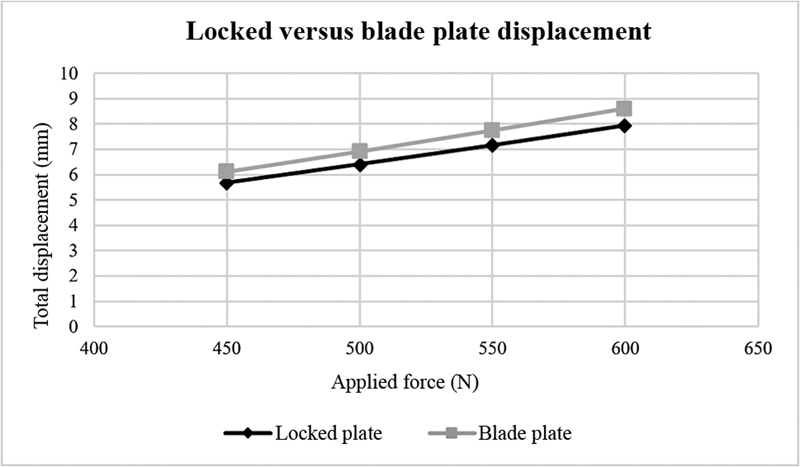
Locked and blade plate displacement versus applied forces. Source: Data constructed by the authors using SolidWorks software.

**Fig. 5:**
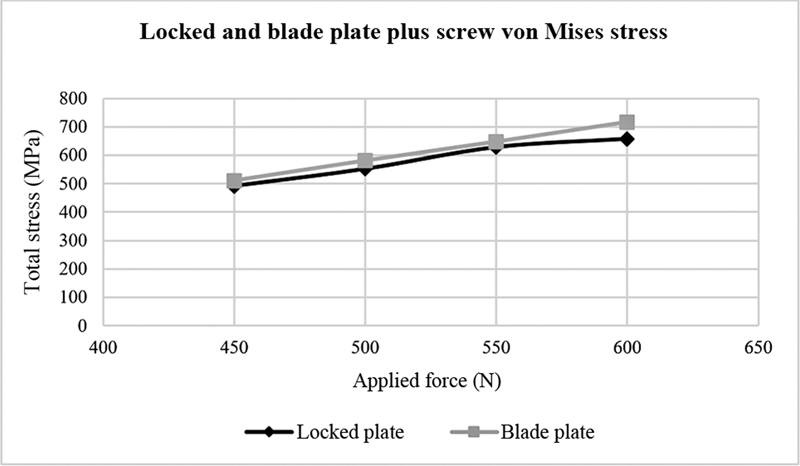
Locked and blade plate von Mises stress versus applied forces. Source: Data constructed by the authors using SolidWorks software.

**Fig. 6:**
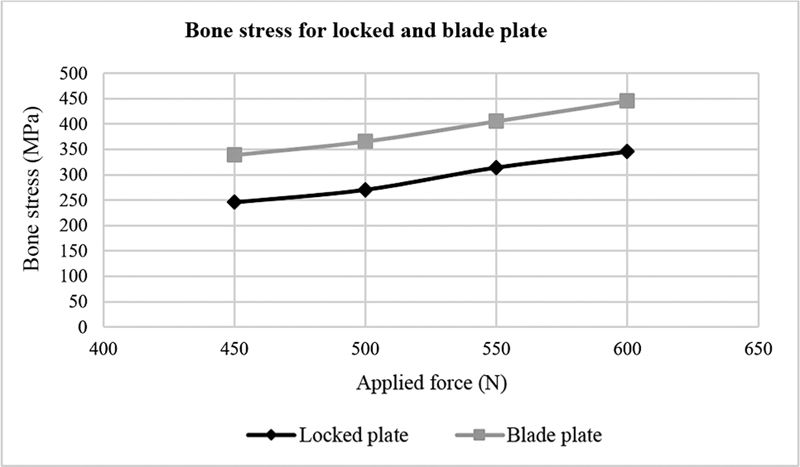
Von Mises stress in the bone versus applied forces. Source: Data constructed by the authors using SolidWorks software.

Von Mises stress and displacement for the blade plate were higher compared with the locked plate ( Tables 2 3 4 to 5 ). Fig. 4 demonstrates the displacement, in millimeters, of locked and blade plates after applying 450, 500, 550, and 600 N forces. Fig. 5 shows the von Mises stress in MPa of locked and blade plates after applying 450, 500, 550, and 600 N forces. Fig. 6 shows the von Mises stress in MPa on the bone fixed with locked or blade plates after applying 450, 500, 550, and 600 N forces.

**Table 2:** von Mises stress and strain for each plate and bone at a 450 N force

Variable	Group	Number of nodes (mesh)	Maximum value	Minimum value	Applied force [N]
**Total displacement [mm]**	**Locked plate**	473,114	5.6797	0	450
	**Blade plate**	325,578	6.1151	0	
**Plate and screw stress [MPa]**	**Locked plate**	473,114	492.1	73.58	
	**Blade plate**	325,578	510.12	93.222	
**Bone stress [MPa]**	**Locked plate**	473,114	245.86	40.469	
	**Blade plate**	325,578	339.41	66.558	

Source: Data obtained by the authors.

**Table 3:** von Mises stress and strain for each plate and bone at a 500 N force

Variable	Group	Number of nodes (mesh)	Maximum value	Minimum value	Applied force [N]
**Total displacement [mm]**	**Locked plate**	473,114	6.4123	0	500
	**Blade plate**	325,578	6.9125	0	
**Plate and screw stress [MPa]**	**Locked plate**	473,114	552.04	82.515	
	**Blade plate**	325,578	580.26	103.87	
**Bone stress [MPa]**	**Locked plate**	473,114	270.61	45.442	
	**Blade plate**	325,578	365.99	73.925	

Source: Data obtained by the authors.

**Table 4:** von Mises stress and strain for each plate and bone at a 550 N force

Variable	Group	Number of nodes (mesh)	Maximum value	Minimum value	Applied force [N]
**Total displacement [mm]**	**Locked plate**	473,114	7.1602	0	550
	**Blade plate**	325,578	7.7405	0	
**Plate and screw stress [MPa]**	**Locked plate**	473,114	628.5	91.55	
	**Blade plate**	325,578	647.06	114.58	
**Bone stress [MPa]**	**Locked plate**	473,114	314.46	50.454	
	**Blade plate**	325,578	405.72	81.292	

Source: Data obtained by the authors.

**Table 5:** von Mises stress and strain for each plate and bone at a 600 N force

Variable	Group	Number of nodes (mesh)	Maximum value	Minimum value	Applied force [N]
**Total displacement [mm]**	**Locked plate**	473,114	7.942	0	600
	**Blade plate**	325,578	8.6027	0	
**Plate and screw stress [MPa]**	**Locked plate**	473,114	657.72	100.69	
	**Blade plate**	325,578	716.19	125.35	
**Bone stress [MPa]**	**Locked plate**	473,114	345.65	55.504	
	**Blade plate**	325,578	445.55	88.664	

Source: Data obtained by the authors.

**Fig. 7:**
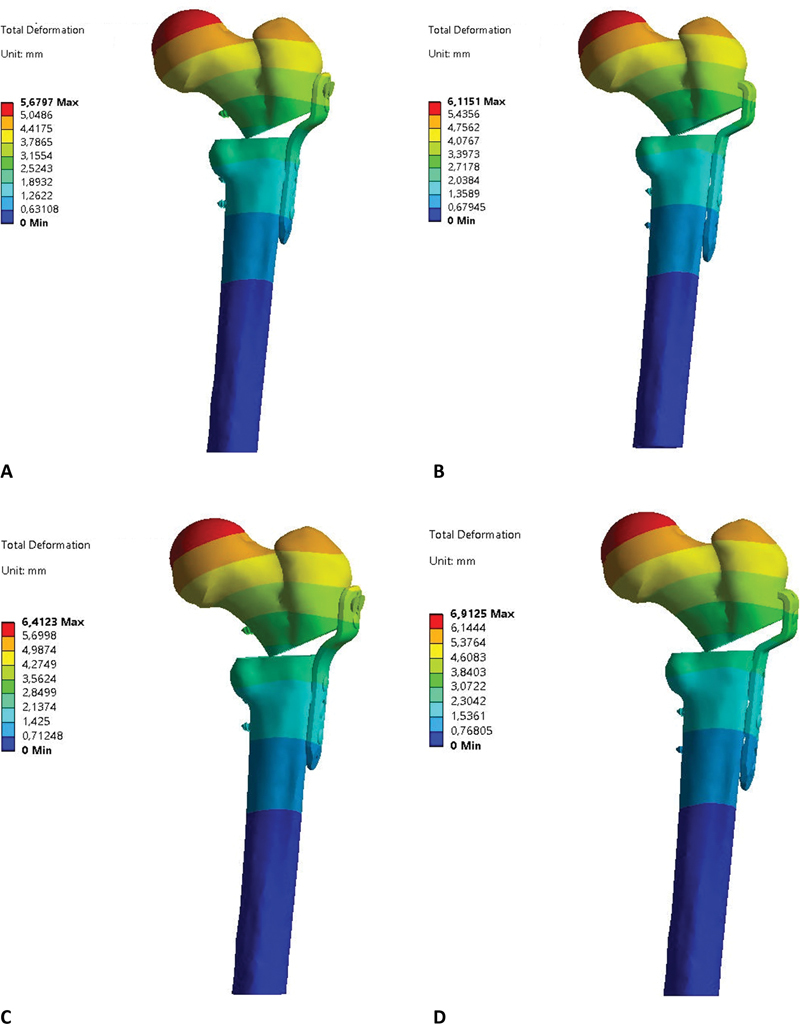
Locked plate total displacement, F = 450 N ( **A** ), blade plate total displacement, F = 450 N ( **B** ), locked plate total displacement, F = 500 N ( **C** ), blade plate total displacement, F = 500 N ( **D** ), locked plate total displacement, F = 550 N ( **E** ), blade plate total displacement, F = 550 N ( **F** ), locked plate total displacement, F = 600 N ( **G** ), blade plate total displacement, F = 600 N ( **H** ). Source: Data constructed by the authors using SolidWorks software.

**Fig. 8:**
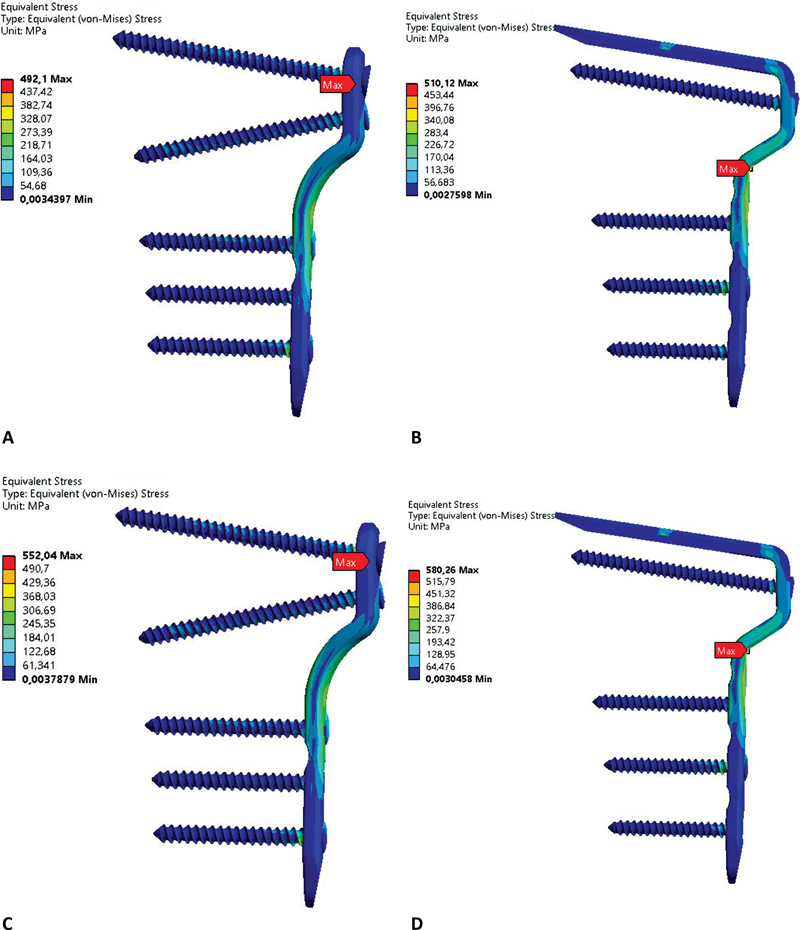
Von Mises stress for the locked plate, F = 450 N ( **A** ), von Mises stress for the blade plate, F = 450 N ( **B** ), von Mises stress for the locked plate, F = 500 N ( **C** ), von Mises stress for the blade plate, F = 500 N ( **D** ), von Mises stress for the locked plate, F = 550 N ( **E** ), von Mises stress for the blade plate, F = 550 N ( **F** ), von Mises stress for the locked plate, F = 600 N ( **G** ), von Mises stress for the blade plate, F = 600 N ( **H** ). Source: Data constructed by the authors using SolidWorks software.

The blade plate caused the highest displacement ( Table 2 ). The highest displacement site for locked and blade plates was at the point of vertical force application at the femoral head ( Fig. 7 ). The blade plate had the highest von Mises stress ( Table 2 ). For the locked plate, the highest stress concentration was in the locking area between the proximal screw and the plate. For the blade plate, the region with the highest stress concentration was at the beginning of the advancement, at the osteotomy level ( Fig. 8 ).

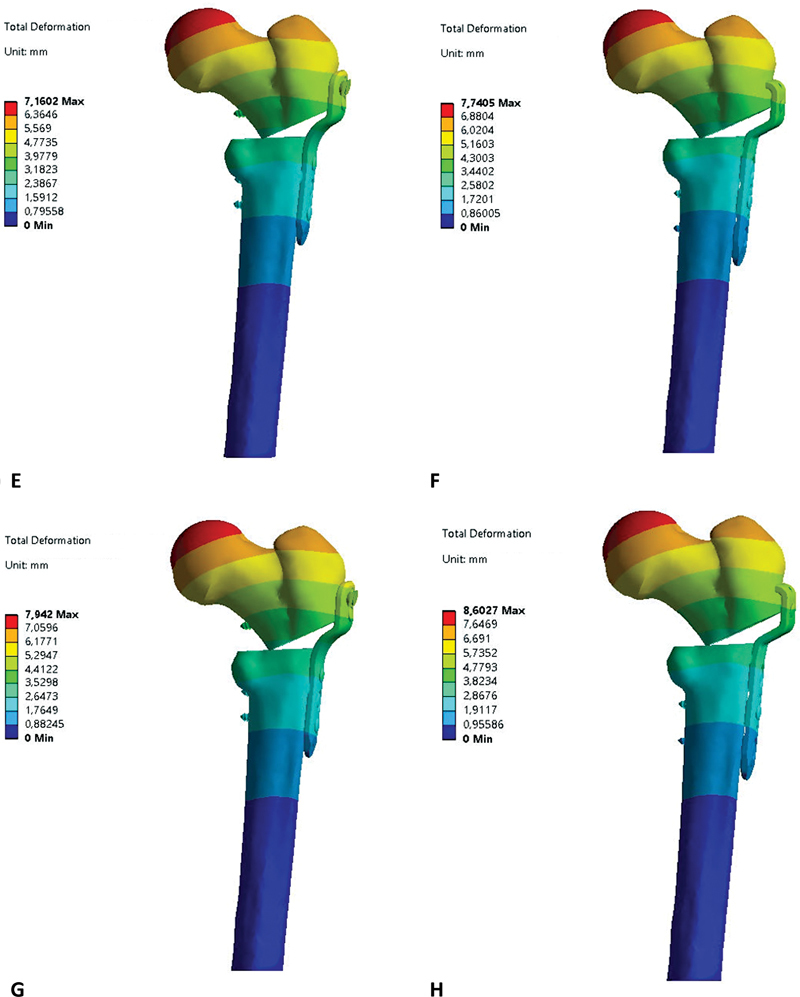



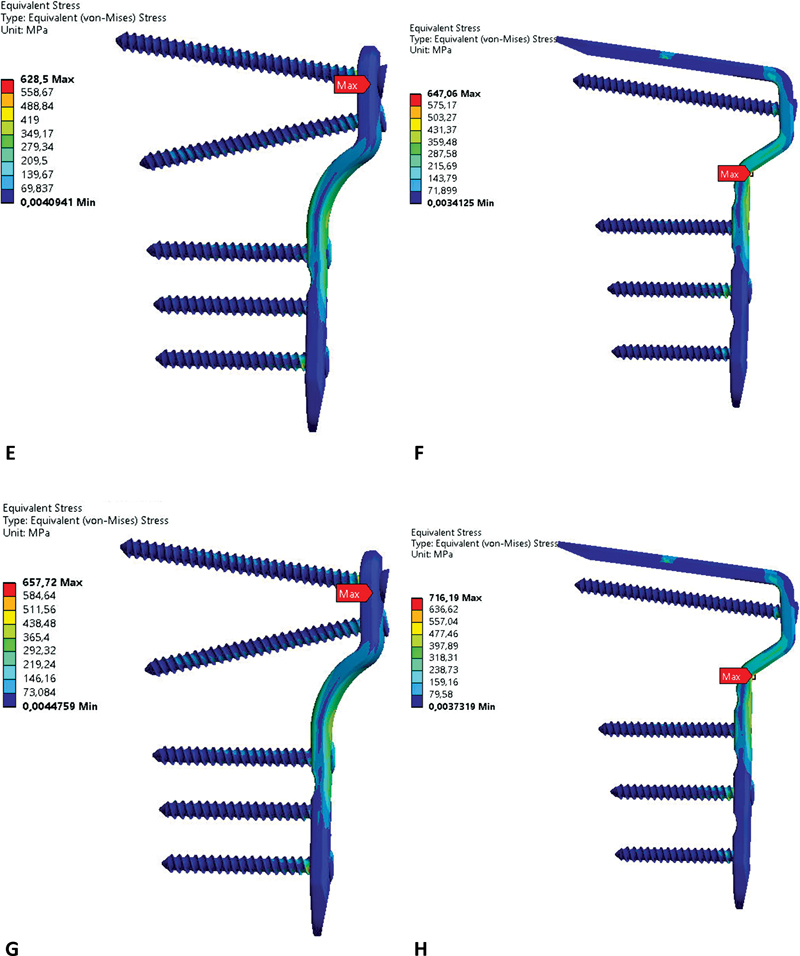



**Fig. 9:**
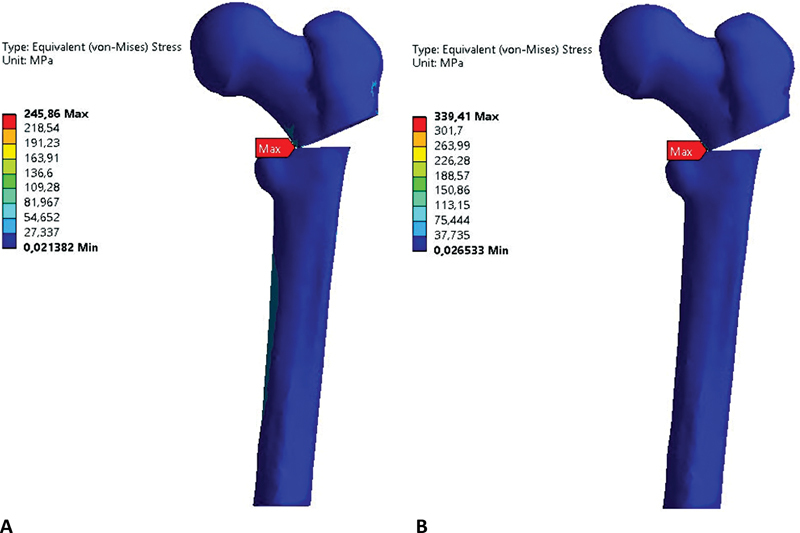
Von Mises stress for locked plate, F = 450 N ( **A** ), von Mises stress for blade plate, F = 450 N ( **B** ). Source: Data constructed by the authors using SolidWorks software.

The bone fixed with the blade plate showed the highest von Mises stress ( Table 2 ). The region with the highest bone stress concentration was the osteotomy, in the corner of the proximal part with the spongy bone surface in the distal part. The stress occurred at the same regions for all applied forces ( Fig. 9 ).

Fixation with the locked plate withstand more cycles compared with the blade plate for loads of 450, 500, 550, and 600 N in a 1 Hz frequency ( Table 6 ).

**Table 6:** Number of cycles for locked and blade plates at a 1 Hz frequency

Applied force [N]	Group	Number of cycles
**450**	**Locked plate**	> 1,000,000
	**Blade plate**	> 1,000,000
**500**	**Locked plate**	617,680
	**Blade plate**	207,710
**550**	**Locked plate**	49,944
	**Blade plate**	32,371
**600**	**Locked plate**	25,362
	**Blade plate**	5,271

Source: Data obtained by the authors.

**Fig. 10:**
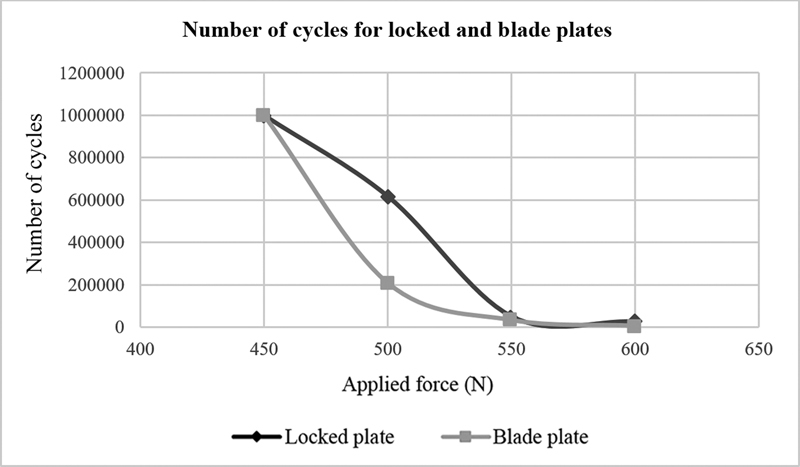
Number of cycles versus applied forces. Source: Data constructed by the authors using SolidWorks software.

Fig. 10 shows the number of cycles for locked and blade plates under 450, 500, 550, and 600 N loads. Furthermore, it demonstrates that after applying a load > 600 N, locked and blade plates tend to fail in the first cycle.

## Discussion

Conduction of clinical investigations on the forces required to stimulate bone healing is complex. The finite element method is widely used in the medical and orthopedic field because it provides a comprehensive view of vector dissolution in undermined structures and allows for accurate failure detection. Moreover, it may avoid unnecessary costs when failure is identifiable only after structural design or manufacturing. The finite element method also reduces the time from the first conceptual design to production, as the creation of a large number of experimental specimens becomes unnecessary. Finite element method analysis provides access to information difficult to obtain under laboratory conditions, such as predicted stress distribution and material strength, which are fundamental to assessing fatigue strength. 18

In the present comparative biomechanical study, we investigated the flexural stiffness of the locked and blade plates for proximal femoral varus osteotomy. Our data suggest the locked plate presents higher stiffness compared with the blade plate. However, this difference was not significant ( Tables 2 3 4 to 5 ). In addition, the locked plate has proximal locking screws to ensure that the bending moments acting on screws are lower during loading.

Locked plate failure occurs with significantly higher forces compared with the blade plate. The locked plate has significantly higher stiffness and load to failure values due to the nature of its design. At 19 mm, the locked plate is 8 mm wider in the proximal area, thus withstanding more stress than the blade plate. Both plates have approximately the same thickness of 3 mm. This results in a higher moment of inertia on the locked plate, which reflects in the proximal force results.

Consistent with our data, a comparative analysis by Radtke et al. 19 found mean values of 554 N for locked plates and 399 N for blade plates. These authors used synthetic bone and plates. Forward et al. 20 reported mean values of 620 N for locked plates and 450 N for blade plates in a study performed with cadavers.

Femoral stress distribution was consistent with a study by Sim et al. 21 reporting a higher stress concentration between the proximal and distal parts at the point of separation osteotomy.

Absolute displacement values were higher for the blade plate, which had two areas of load application with higher variation (femur head). However, there is a biomechanical advantage associated with the stress areas of the system. In locked plates, the stress area of the joint between the plate and the bone is at the proximal screw. On the other hand, in blade plates, this area is at the blade advance region. Thus, in blade plates, all the stress concentrates in the osteotomy region, which usually constitutes an obstacle to bone consolidation resulting from the Wolff law. 22 The success of biological bone healing depends on a favorable mechanical environment. In addition, the Wolff law and the Perren strain theory allow using several osteosynthesis systems to promote adequate stabilization and the differentiation of various cell types at the bone healing site. 22 23

The relative stability indicated for comminuted diaphyseal or extra-articular fractures allows for some controlled mobility at the fracture site and exuberant formation of bone callus, which characterizes an indirect or endochondral ossification. Direct or intramembranous ossification is recommended to avoid developing bulky bone calluses in joint fractures, following absolute fixation with greater rigidity. 24

The locked plate system also presents a larger stress area at the osteotomy region but produced lower absolute values than the blade plate. Thus, we inferred that locked plates create a more favorable biomechanical situation for bone consolidation.

Our study has limitations regarding FEM, which considers structures as a gathering of small particles of finite quantity, the so-called finite elements, connected to a finite number of points, the nodes, or nodal points. These particles represent the approximate result of every discretized system. 25 The finite element method allows the evaluation of the approximate stress distribution in a structure, observing the element strain through visualization and image interpretation on a color chart. 5

The present study demonstrated that the blade plate resisted fewer cycles when the loading forces were lower. However, locked and blade plates tend to fail under the highest applied force. Thus, despite being exposed to controlled load situations, the locked plate was more resistant to implant failure. In exacerbated load situations, locked and blade plates tend to fail.

Both osteosynthesis implants are consecrated and effective. Nevertheless, our study using FEM shows a fundamental role in understanding the biomechanical situation of the implant. It also opens up new possibilities for further studies, including surgical proposals and customized implant materials. Therefore, our study corroborates a hypothesis raised by common sense, that is, the superiority of the locked plate compared with blade plates for varus osteotomy of the proximal femur. Still, there is no unanimity in the literature, especially regarding clinical outcomes.

Our study may yield future models with laboratory biomechanical tests to prove the differences between locked and blade plate fixation systems.

## Conclusion

Both osteosynthesis implants are well-established and effective in clinical practice. However, our study applying FEM demonstrated the biomechanical superiority of the locked plate compared with the blade plate for proximal femoral varus osteotomy in the proposed model.
