# Supplementary material for: Comparison of Locked Plates and Blade Plates for Varus Osteotomy of the Proximal Femur by the Finite Element Method
Source: Rev Bras Ortop (Sao Paulo). 2024 Mar 21;59(1):e107–18. doi: 10.1055/s-0043-1775889 (PMC10957266; doi:10.1055/s-0043-1775889)
Supplement: Related texts

# Research article: Avaliação entre placas bloqueadas e placas lâmina para osteotomia varizante do fêmur proximal pelo método dos elementos finitos

**Keywords:** ortopedia, traumatologia, fêmur, placas ósseas

## Abstract

**Objetivo:** Testar a diferença do comportamento de distribuição de cargas e pressões da placa lâmina comparativamente com a placa bloqueada para osteotomia varizante do fêmur proximal através do método de elementos finitos.

**Métodos:** A modelagem foi realizada através do escaneamento de um fêmur esquerdo com deformidade medial em valgo fabricado em poliuretano de tamanho médio.

**Resultados:** Como resultados, pode-se inferir que a rigidez da placa bloqueada é maior do que a da placa lâmina. No entanto, essa diferença não foi significativa e, além disso, a placa bloqueada possui parafusos de travamento proximal para garantir que os momentos de flexão que agem nos parafusos sejam ainda menores durante o carregamento.

**Conclusão:** Em síntese, ambos os materiais são consagrados e eficazes para serem utilizados, porém o estudo pelo método de elementos finitos apresenta papel importante para compreendermos a situação de distribuição de cargas e pressões do implante e abre novas possibilidades para novos estudos, como, por exemplo, o estudo da proposta cirúrgica e materiais a serem implantados de forma individual e personalizada.

## Introdução

As osteotomias varizantes do fêmur proximal são procedimentos cirúrgicos reconstrutivos pediátricos amplamente realizados em pacientes com diagnóstico de anormalidades neurológicas, doenças congênitas do quadril, sequelas e patologias adquiridas. 1 2 Diversos implantes são utilizados para a fixação destas osteotomias, tais como: placas lâmina; placas de compressão dinâmica (DCP, na sigla em inglês); placas bloqueadas para fêmur proximal; fixadores externos unilaterais e circulares, fios de Kirschner e parafusos. 1 2 3 Devido à grande dificuldade cirúrgica para a realização deste procedimento, os materiais de síntese envolvidos vêm sendo aprimorados para facilitar e melhorar seus resultados. 1 2 4

Dentre os principais materiais utilizados, destaca-se a placa bloqueada e as placas lâmina para a osteotomia varizante do fêmur proximal. 1 2 3 4 Clinicamente, não há diferença estatística quanto ao risco de falha (quebra) entre as placas lâmina e as bloqueadas, 3 sendo esta considerada a pior complicação relacionada à escolha do implante. Testes biomecânicos de aplicação de carga em modelos experimentais apontam que a placa bloqueada com parafuso de suporte apresenta maior resistência axial, menor resistência à torção e equivalente deformação irreversível aos ciclos deformantes quando comparadas à placa lâmina. 4

O método de elementos finitos (MEF) é uma ferramenta matemática utilizada para resolver problemas em engenharia, sendo capaz de explorar os efeitos da aplicação de uma carga no osso e seu comportamento biomecânico. Uma de suas vantagens principais é poder ser utilizado em sólidos de geometria irregular que contenham propriedades materiais heterogêneas. O MEF foi introduzido na biomecânica ortopédica a partir da década de 1970 e, desde então, tem sido crescente o número de publicações de estudos de análise de carregamento em ossos, artroplastias e osteossínteses. 5

O objetivo do presente estudo é analisar pelo MEF o comportamento na distribuição de cargas e pressões entre o uso da placa lâmina e da placa bloqueada para osteotomia varizante do fêmur proximal.

## Método

## Geração do Modelo

A modelagem foi feita através do escaneamento de um fêmur esquerdo por tomografia computadorizada com deformidade medial em valgo fabricado em poliuretano de tamanho médio da marca Nacional Ossos (ref. 2025 DMVL). O software utilizado para escaneamento e modelagem das placas foi o SolidWorks (Dassault Systems SolidWorks Corp., Waltham, MA, EUA) ( Fig. 1a ).

**Fig. 1:** Fêmur valgo para escaneamento Nacional Ossos. ( **A** ) fêmur simplificado e ( **B** ) osteotomia varizante. Fonte: Dados construídos pelos autores com o *software SolidWorks* .

A simplificação do fêmur foi feita através do corte na diáfise descartando a parte distal, que não será contemplada no presente estudo. Também já foi realizada a osteotomia varizante para correção de 20°. Esta simplificação proporcionou um ganho em processamento do cálculo pelo método dos elementos finitos, sem ocasionar distorção nos resultados. 6 O ambiente (fêmur) será o mesmo para ambos os estudos, não distorcendo ou favorecendo os resultados obtidos ( Fig. 1b ).

O primeiro fêmur foi fixado com a placa bloqueada para fêmur proximal, passo de 10 mm, 100°, com 3 furos (Techimport, Rio Claro, SP, Brasil) (ref. TI030.1003.100). Foram utilizados no fragmento proximal parafusos bloqueados, com diâmetro de Ø3,5 × 50 mm em dois furos e Ø3,5 × 40 mm no terceiro furo. No fragmento distal, foi utilizado inicialmente um parafuso não bloqueado de compressão no furo da placa com diâmetro de Ø3,5 × 30 mm e dois parafusos bloqueados de Ø3,5 × 30 mm ( Fig. 2a ). O segundo fêmur foi fixado com a placa lâmina para fêmur proximal, passo de 10 mm, 100°, com 3 furos, lâmina de 50mm, Techimport, Rio Claro, SP, Brasil ^®^ (ref. TI030.1010.350). Foram utilizados parafusos bloqueados, com diâmetro de Ø3,5 × 50mm no furo proximal, dois parafusos não bloqueados de compressão nos furos não bloqueados distais da placa com diâmetro de Ø3,5 × 30mm e um parafuso bloqueado de Ø3,5 × 30mm para fixação distal ( Fig. 2b ).

**Fig. 2:** Placa bloqueada fixada no fêmur com parafusos ( **A** ). Placa lâmina fixada no fêmur com parafusos ( **B** ). Fonte: Dados construídos pelos autores com o *software SolidWorks* .

A montagem das placas e parafusos foi feita através do posicionamento visual utilizando o software SolidWorks (Dassault Systems SolidWorks Corp., Waltham, MA, EUA). A restrição de distância de 27 mm entre o eixo do parafuso proximal da placa bloqueada e eixo central da placa lâmina, assim como a restrição de distância de 71 mm entre a face medial do fêmur proximal e a face interna das placas, foram criadas para padronizar o momento flexor gerado pela aplicação da força axial e avaliar a tensão de deslocamento gerada ( Fig.2 ).

## Propriedades dos Materiais

Os modelos de placas, parafusos e ossos corticais e esponjosos foram homogêneos, lineares, elásticos e isotrópicos, modelados conforme propriedades obtidas na literatura ( Tabela 1 ). O valor de 795 MPa foi utilizado para tensão de escoamento, que é a tensão limite do material na região elástica da liga de titânio. 7 8

**Tabela 1:** Módulo elástico, razão de Poisson e número de ciclos da fadiga de todos os materiais (valores de Maurer et al., 1999, 5 M. Janecek et al., 2015 6 )

Materiais	Módulo de elasticidade (E) [MPa]	Coeficiente de Poisson	Tensão de escoamento a 10 ^6^ ciclos [MPa]
Osso Cortical	8.700	0,33	200
Osso Esponjoso	500	0,30	125
Liga de Titânio	110.000	0,34	540

Fonte: Dados obtidos pelos autores.

## Parâmetros de Simulação (Carga, Malha e Condições de Contato)

Conforme apresentado na Fig. 2 , as forças aplicadas foram de 450, 500, 550 e 600N, considerada a posição de carregamento normal, que assume que o vetor de carga possui um ângulo de 8° aduzido com o eixo longitudinal do quadril no plano. 6 9 10 11 12 13 14 15 16 17 Em uma situação clínica, ambos os valores são maiores do que os produzidos pelo apoio de toque com muletas e, portanto, devem fornecer estabilidade pós-operatória suficiente. 10 11

O valor da frequência de aplicação da força foi 1 Hz, ou seja, considerando uma caminhada de 1 passo por segundo. 11 12 Com este valor, a placa deve suportar no mínimo 10 ^6^ ciclos, por se tratar de um dispositivo de fixação temporária, o equivalente a ∼ 1 ano. 13 A face transversal da diáfise do fêmur foi restringida em todos os graus de liberdade de translação ( Fig. 2 ).

Os modelos de malhas foram mesclados no software SolidWorks com elementos tetraédricos quadráticos tridimensionais de 1 mm para placas, 0,5 mm para os parafusos e 5 mm para o osso. 14 O contato de ligação foi definido como ocorrendo entre o tecido ósseo e os implantes, com exceção das interfaces da osteotomia ( Fig. 3 ).

**Fig. 3:** Malha aplicada aos modelos tridimensionais da placa bloqueada ( **A** ). Malha aplicada aos modelos tridimensionais da placa lâmina ( **B** ). Fonte: Dados construídos pelos autores com o *software SolidWorks* .

Para as superfícies de contato entre placa e parafuso, foi considerado um coeficiente de atrito de 0,34. 15 17 O contato entre o parafuso e placa foi através da superfície da cabeça do parafuso e a superfície do escareado nos furos da placa, todos com a restrição para não permitir penetração entre eles. Já para os contatos entre os parafusos bloqueados nos furos das placas, foram considerados como ligados e fixos. 17

## Resultados

Pode ser verificado que os valores de tensão de von-Mises e deslocamento para placa lâmina foram maiores que os valores da placa bloqueada ( Tabelas 2 3 4 a 5 ). A Fig. 4 demonstra o deslocamento em mm da placa bloqueada e da lâmina, com a aplicação de forças nos valores de 450, 500, 550 e 600N. A Fig. 5 demonstra a tensão de von-Mises em MPa da placa bloqueada e da lâmina, com a aplicação de forças nos valores de 450, 500, 550 e 600N. A Fig. 6 demonstra a tensão de von-Mises em MPa no osso quando fixado com a placa bloqueada ou com a lâmina, com a aplicação de forças nos valores de 450, 500, 550 e 600N.

**Tabela 2:** Deformação e Tensão de von-Mises para cada modelo de placa e osso para força de 450N

Variável	Grupo	Número de nós (malha)	Valor máximo	Valor mínimo	Força aplicada [N]
**Deslocamento total [mm]**	**Bloqueada**	473.114	5,6797	0	450
	**Lâmina**	325.578	6,1151	0	
**Tensão placa e parafusos [MPa]**	**Bloqueada**	473.114	492,1	73,58	
	**Lâmina**	325.578	510,12	93,222	
**Tensão osso [MPa]**	**Bloqueada**	473.114	245,86	40,469	
	**Lâmina**	325.578	339,41	66,558	

Fonte: Dados obtidos pelos autores.

**Tabela 3:** Deformação e Tensão de von-Mises para cada modelo de placa e osso para Força de 500N

Variável	Grupo	Número de nós (malha)	Valor máximo	Valor mínimo	Força aplicada [N]
**Deslocamento total [mm]**	**Bloqueada**	473.114	6,4123	0	500
	**Lâmina**	325.578	6,9125	0	
**Tensão placa e parafusos [MPa]**	**Bloqueada**	473.114	552,04	82,515	
	**Lâmina**	325.578	580,26	103,87	
**Tensão osso [MPa]**	**Bloqueada**	473.114	270,61	45,442	
	**Lâmina**	325.578	365,99	73,925	

Fonte: Dados obtidos pelos autores.

**Tabela 4:** Deformação e Tensão de von-Mises para cada modelo de placa e osso para Força de 550N

Variável	Grupo	Número de nós (malha)	Valor máximo	Valor mínimo	Força aplicada [N]
**Deslocamento Ttotal [mm]**	**Bloqueada**	473.114	7,1602	0	550
	**Lâmina**	325.578	7,7405	0	
**Tensão placa e parafusos [MPa]**	**Bloqueada**	473.114	628,5	91,55	
	**Lâmina**	325.578	647,06	114,58	
**Tensão osso [MPa]**	**Bloqueada**	473.114	314,46	50,454	
	**Lâmina**	325.578	405,72	81,292	

Fonte: Dados obtidos pelos autores.

**Tabela 5:** Deformação e Tensão de von-Mises para cada modelo de placa e osso para Força de 600N

Variável	Grupo	Número de nós (malha)	Valor máximo	Valor mínimo	Força aplicada [N]
**Deslocamento total [mm]**	**Bloqueada**	473.114	7,942	0	600
	**Lâmina**	325.578	8,6027	0	
**Tensão placa e parafusos [MPa]**	**Bloqueada**	473.114	657,72	100,69	
	**Lâmina**	325.578	716,19	125,35	
**Tensão osso [MPa]**	**Bloqueada**	473.114	345,65	55,504	
	**Lâmina**	325.578	445,55	88,664	

Fonte: Dados obtidos pelos autores.

**Fig. 4:** Deslocamento da placa bloqueada e lâmina *versus* forças aplicadas. Fonte: Dados construídos pelos autores com o *software SolidWorks* .

**Fig. 5:** Tensão de von-Mises da placa bloqueada e lâmina *versus* forças aplicadas. Fonte: Dados construídos pelos autores com o *software SolidWorks* .

**Fig. 6:** Tensão de von-Mises no osso *versus* forças aplicadas. Fonte: Dados construídos pelos autores com o *software SolidWorks* .

A placa lâmina apresentou os maiores valores de deslocamento ( Tabela 2 ). O maior deslocamento de ambas ficou localizado no ponto de aplicação da força vertical, na cabeça do fêmur ( Fig. 7 ). A placa lâmina apresentou os maiores valores de tensão de von-Mises ( Tabela 2 ). A região com maior concentração de tensão da placa bloqueada ficou localizada no bloqueio entre o parafuso proximal e a placa. Já a região com maior concentração de tensão da placa lâmina ficou localizada no início do avanço, na altura da osteotomia ( Fig. 8 ).

**Fig. 7:** Deslocamento total da placa bloqueada F = 450N ( **A** ), Deslocamento total da placa lâmina F = 450N ( **B** ), Deslocamento total da placa bloqueada F = 500N ( **C** ), Deslocamento total da placa lâmina F = 500N ( **D** ), Deslocamento total da placa bloqueada F = 550N ( **E** ), Deslocamento total da placa lâmina F = 550N ( **F** ), Deslocamento total da placa bloqueada F = 600N ( **G** ), Deslocamento total da placa lâmina F = 600N ( **H** ). Fonte: Dados construídos pelos autores com o *software SolidWorks* .



**Fig. 8:** Tensão de von-Mises da placa bloqueada F = 450N ( **A** ), Tensão de von-Mises da placa lâmina F = 450N ( **B** ), Tensão de von-Mises da placa bloqueada F = 500N ( **C** ), Tensão de von-Mises da placa lâmina F = 500N ( **D** ), Tensão de von-Mises da placa bloqueada F = 550N ( **E** ), Tensão de von-Mises da placa lâmina F = 550N ( **F** ), Tensão de von-Mises da placa bloqueada F = 600N ( **G** ), Tensão de von-Mises da placa lâmina F = 600N ( **H** ). **Fonte:** Dados construídos pelos autores com o *software SolidWorks* .



O osso fixado com a placa lâmina apresentou os maiores valores de tensão de von-Mises ( Tabela 2 ). A região com maior concentração de tensão do osso ficou localizada na osteotomia, no canto da parte proximal com a superfície esponjosa do osso na parte distal. Em todas as aplicações de força, a tensão ficou localizada na mesma região ( Fig. 9 ).

**Fig. 9:** Tensão de von-Mises no osso para placa bloqueada F = 450N ( **A** ), Tensão de von-Mises no osso para placa lâmina F = 450N ( **B** ). Fonte: Dados construídos pelos autores com o *software SolidWorks* .

A fixação com a placa bloqueada apresentou um número de ciclos maior do que a fixação com a placa lâmina, para os carregamentos de 450, 500, 550 e 600N com uma frequência de 1 Hz ( Tabela 6 ).

**Tabela 6:** Número de ciclos nas placas bloqueada e lâmina com frequência de 1Hz

Força Aplicada [N]	Grupo	Número de Ciclos
**450**	**Bloqueada**	> 1.000.000
	**Lâmina**	>1.000.000
**500**	**Bloqueada**	617.680
	**Lâmina**	207.710
**550**	**Bloqueada**	49.944
	**Lâmina**	32.371
**600**	**Bloqueada**	25.362
	**Lâmina**	5.271

Fonte: Dados obtidos pelos autores.

A Fig. 10 demonstra o número de ciclos das placas bloqueada e da lâmina para carregamento nos valores de 450, 500, 550 e 600N, demonstrando, ainda, que após a aplicação de um carregamento > 600N para placa bloqueada ou lâmina a tendência é que ambas falhem no primeiro ciclo.

**Fig. 10:** Número de ciclos *versus* forças aplicadas. Fonte: Dados construídos pelos autores com o *software SolidWorks* .

## Discussão

Dada a dificuldade de realizar investigações clínicas sobre as forças necessárias para estimular a cicatrização óssea, o MEF tem sido amplamente utilizado na área médica e ortopédica, pois fornece uma visão abrangente da dissolução de vetores em estruturas solapadas, detecção precisa de falhas, e ainda evita custos desnecessários nos casos em que a falha só seria identificada após o projeto estrutural ou fabricação. Além disso, o tempo desde o primeiro projeto conceitual até a produção é reduzido, pois a fabricação de um grande número de corpos de prova experimentais torna-se desnecessária. A análise de elementos finitos fornece acesso a informações que são muito difíceis de obter em condições de laboratório, como a distribuição de tensão prevista e resistência do material que são de grande importância na avaliação da resistência à fadiga. 18

No presente estudo biomecânico comparativo, investigamos a rigidez flexional entre placa bloqueada e a placa lâmina para osteotomia varizante de fêmur proximal. Pode-se inferir à luz dos dados obtidos que a rigidez da placa bloqueada é maior do que a da placa lâmina. No entanto, essa diferença não foi significativa ( Tabelas 2 3 4 a 5 ). Além disso, a placa bloqueada possui parafusos de travamento proximal para garantir que os momentos de flexão que agem nos parafusos sejam ainda menores durante o carregamento.

A falha do implante ocorre apenas com forças significantemente maiores na placa bloqueada quando comparada com a placa lâmina. É correto afirmar que a placa bloqueada tenha uma rigidez e “carga até a falha” significativamente maiores, devido à natureza da concepção da mesma. Com 19 mm, a placa bloqueada é 8 mm mais larga na área proximal, suportando, assim, mais tensão do que a placa lâmina. Ambas as placas têm aproximadamente a mesma espessura, de 3 mm. Isso resulta em um maior momento de inércia na placa bloqueada, refletindo-se nos resultados da força proximal.

Validando os valores encontrados no presente estudo, a análise comparativa de Radtke et al. 19 encontrou valores médios de 554 N para placa bloqueada e 399 N para placa lâmina; tal estudo foi realizado com osso sintético e placas. Em outro estudo, Forward et al. 20 também encontraram valores médios de 620 N para placa bloqueada e 450 N para placa lâmina; tal estudo foi realizado com cadáver e placas.

A distribuição de tensão no fêmur apresentado nesse estudo ratifica o estudo de Sim et al., 21 demonstrando maior concentração de tensões entre as partes proximal e distal no ponto da osteotomia de separação.

Os valores absolutos de deslocamento foram maiores para a placa lâmina, tendo as duas áreas de aplicação da carga com maior variação desta (cabeça do fêmur). Tem-se, porém, a vantagem biomecânica associada às áreas de tensão do sistema, na placa bloqueada; a área de tensão do conjunto entre a placa e o osso é a região do parafuso proximal. Em contrapartida, na placa lâmina, ela se encontra na região do avanço da lâmina. Assim, na placa lâmina, temos a tensão toda concentrada na região da osteotomia, que, de forma geral, é um obstáculo para a consolidação óssea levando em conta as Leis de Wolf. 22 O sucesso da consolidação óssea biológica parece depender de um ambiente mecânico favorável e, sob a lei de Wolff e a teoria da deformação de Perren, vários sistemas de osteossíntese podem ser usados para promover a estabilização adequada e diferentes tipos de diferenciação celular no local da fratura. 22 23

A relativa estabilidade indicada para fraturas diafisárias ou extra-articulares cominutivas permite certa mobilidade controlada no local da fratura e formação exuberante de calo ósseo, o que caracteriza uma ossificação indireta ou endocondral. Para evitar a formação de calos ósseos volumosos nas fraturas articulares, recomenda-se a ossificação direta ou intramembranosa seguindo a fixação absoluta com maior rigidez. 24

O sistema com placa bloqueada também apresenta uma maior área de tensão na região da osteotomia; porém, possui valores absolutos menores do que os da placa lâmina. Desta forma, pode-se inferir uma situação biomecânica mais favorável para a consolidação óssea com uso da placa bloqueada.

Nosso estudo apresenta limitações com a utilização do MEF, que considera as estruturas como sendo uma reunião de pequenas partículas de quantidade finita, denominadas de elementos finitos, conectados a um número finito de pontos, chamados de nós ou pontos nodais. Tais partículas representam o resultado aproximado de todo sistema discretizado. 25 Por meio do MEF, pode-se avaliar a distribuição das tensões aproximada em uma estrutura, observando a deformação no elemento ou visualizando e interpretando as imagens, através de um gráfico de cores. 5

Nosso estudo demonstrou que a placa lâmina resistiu a menos ciclos quando as forças de carregamento foram menores, apesar de ambas apresentarem tendência a falharem com a maior força aplicada. Dessa forma, apesar de exposta a situações controladas de carga, a placa bloqueada mostrou-se mais resistente à falha do implante. Em situações de carga exacerbada, ambas tendem a ceder.

Ambos os implantes de osteossíntese são consagrados e eficazes para serem utilizados; entretanto, nosso estudo pelo método dos elementos finitos demonstra um papel fundamental para compreendermos a situação biomecânica do implante e abre novas possibilidades para novos estudos, como, por exemplo, o estudo da proposta cirúrgica e materiais a serem implantados de forma individual e personalizada. Neste caso, o presente estudo foi responsável por corroborar uma hipótese já levantada pelo senso comum: a superioridade da placa bloqueada diante das placas lâmina para a osteotomia varizante do fêmur proximal. Contudo, não existe unanimidade na literatura, principalmente quanto aos resultados clínicos.

A partir do presente estudo, podemos derivar novos modelos futuros com testes biomecânicos realizados em laboratório para comprovar as diferenças entre os sistemas de fixação de placa bloqueada *versus* placa lâmina.

## Conclusão

Ambos os implantes de osteossíntese são consagrados e eficazes para serem utilizados na prática clínica; entretanto, nosso estudo aplicando o método dos elementos finitos demonstrou uma superioridade no comportamento biomecânico da placa bloqueada diante das placas lâmina para a osteotomia varizante do fêmur proximal no modelo proposto.
